# Clinical utility of nanopore-targeted sequencing for diagnosing and treating pulmonary infectious diseases from bronchoalveolar lavage fluid

**DOI:** 10.3389/fcimb.2025.1469440

**Published:** 2025-02-19

**Authors:** Gen Li, He Sun, Yangqin Ye, Liqiong Chen, Wenyan Zhang, Shanshan Yu, Qiang Li, Lieying Fan

**Affiliations:** ^1^ Department of Clinical Laboratory, Shanghai East Hospital, Tongji University School of Medicine, Shanghai, China; ^2^ Department of Respiratory and Critical Care Medicine, Shanghai East Hospital, Tongji University School of Medicine, Shanghai, China

**Keywords:** conventional microbial testing, nanopore-targeted sequencing, pulmonary infectious diseases, bronchoalveolar lavage fluid, diagnostic

## Abstract

**Background:**

Conventional microbial testing (CMTs) for infectious pathogens faces challenges in rapid and comprehensive detection. Nanopore-targeted sequencing (NTS) is a novel approach for rapid identification of pathogens; however, clinical experience with the application of NTS is limited.

**Methods:**

We evaluated the diagnostic value of NTS for detecting microbes in bronchoalveolar lavage fluid samples in patients with pulmonary infectious disease (PID, 137 cases), non-pulmonary infectious disease (NPID, 32 cases), or with an unknown etiology (11 cases). We performed a comparative analysis of the diagnostic efficacy of NTS and CMTs in identifying pulmonary infectious diseases and investigated the clinical utility of NTS as a diagnostic tool.

**Results:**

NTS was significantly more sensitive than CMTs in detecting PID (86.13% vs 67.15%, P < 0.01), particularly for important specific pathogens. There were no significant differences between NTS and CMTs in terms of specificity, positive predictive value or negative predictive value. Moreover, NTS (not CMTs) detected 56 microorganisms consistent with clinical presentation, indicating that NTS can provide clinicians with additional support for infection diagnosis. Additionally, prior antibiotic exposure had no influence on the detection efficiency of NTS but significantly hindered that of CMTs. After antibiotic adjustments based on NTS findings, 87.76% of patients showed significant improvement, with a notable decrease in the level of inflammatory markers (CRP, NP, PCT, WBC) post-treatment. Furthermore, NTS can significantly shorten turnaround time and provide real-time results for rapid decision making.

**Conclusions:**

NTS is more efficient than CMTs in diagnosing pulmonary infectious diseases, particularly in detecting critical or specific pathogens, providing faster and more accurate clinical information even for patients with prior antibiotic exposure. Moreover, NTS can assist clinicians in formulating more effective anti-infection strategies.

## 1 Introduction

Pulmonary infectious diseases (PID) is a complex and common respiratory tract infectious disease with high morbidity and mortality rates, especially in older and immunocompromised individuals (Magill et al., 2014; Claassen-Weitz et al., 2021). PID can be caused by a variety of pathogens, including bacteria, viruses, fungi, and parasites. Rapid and accurate identification of respiratory pathogens is critical to the management and treatment of patients with respiratory infections (Miao et al., 2018). Currently, conventional methods for diagnosing infectious pathogens, including microbial culture, antigen/antibody testing, and PCR assays, have limited ability to identify a broad range of pathogens or provide timely results, thus hindering clinical decision making (Huffnagle et al., 2017). Metagenomic next-generation sequencing (mNGS) has been implemented in current clinical practice and has the capability of detecting almost all pathogens present, compared to conventional microbial testing (CMTs). However, the sensitivity of mNGS can be influenced by host and environmental species. Further, short sequencing read length requires higher sequencing depth and more complicated bioinformatics processing, causing a delay in the clinician receiving the final report (Petersen et al., 2019). These factors limit the widespread clinical application of mNGS. In contrast, nanopore-targeted sequencing (NTS) effectively addresses the limitations of mNGS via targeted amplification of pathogens and by providing longer sequencing read lengths with more information (Fu et al., 2022). Recent studies have reported the effectiveness of NTS in the diagnosis of bloodstream infections and infectious endophthalmitis (Hong et al., 2023; Huang et al., 2021; Zhang et al., 2023). In this research, we aim to retrospectively analyze and compare the diagnostic value of NTS with CMTs in bronchoalveolar lavage fluid (BALF) from patients clinically diagnosed with pulmonary infectious diseases, and explore the application of NTS in guiding clinical treatment for PID.

## 2 Materials and methods

### 2.1 Patients

**Figure 1:**
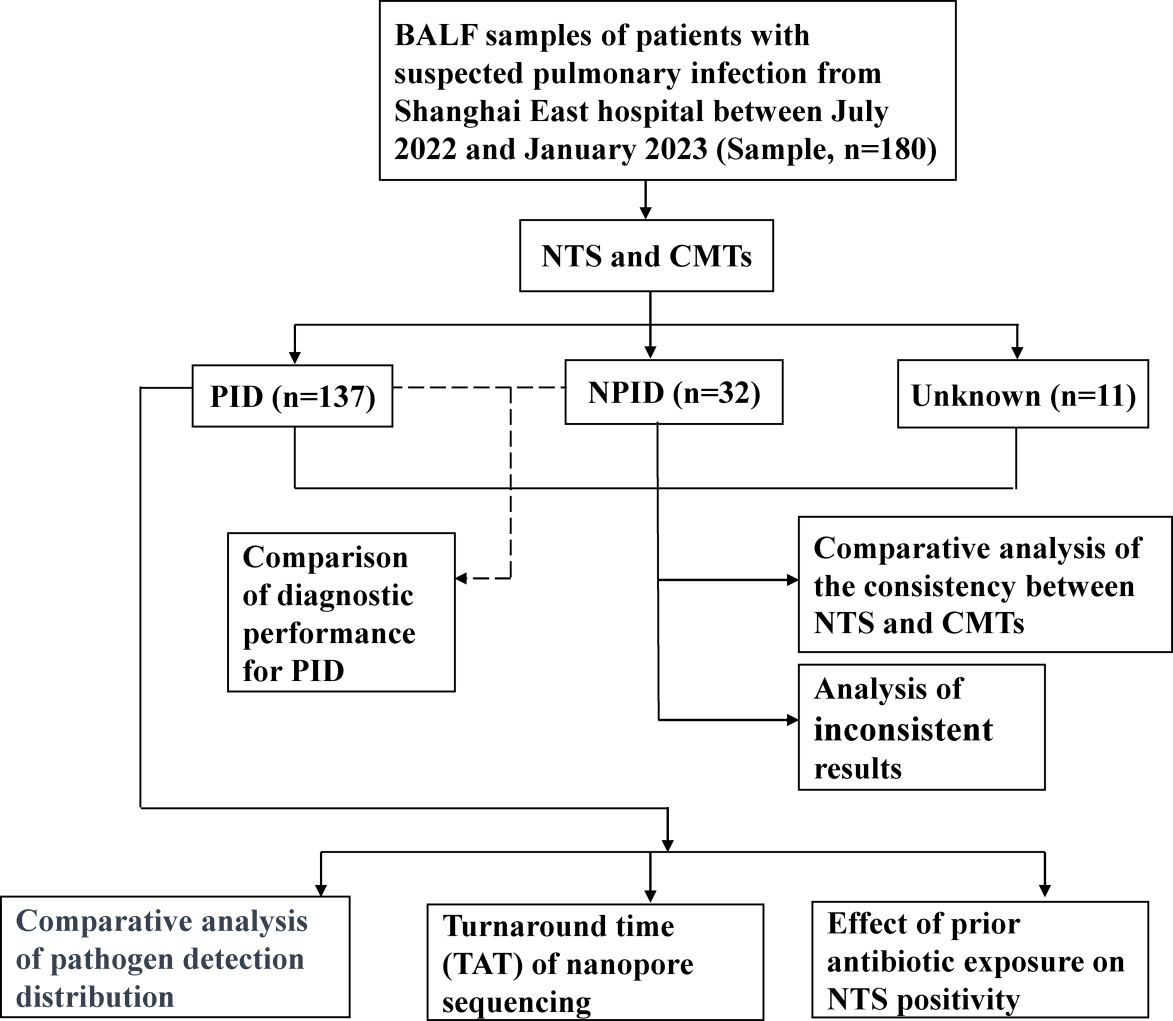
Flowchart of the clinical validation study.

Between July 2022 and January 2023, we retrospectively reviewed 180 cases of initially suspected PID cases at the East Hospital in Shanghai, China. All patients underwent bronchoscopy to collect BALF samples. Samples were immediately prepared for NTS and CMTs. The concordance between NTS and CMTs was analyzed for all samples, and diagnostic performance was assessed using PID and NPID samples. Further comparative analysis was performed on the distribution of pathogens detected by NTS and CMTs in the PID group, as well as an exploration of the potential clinical benefits of NTS (**Figure 1**). This study was approved by the Ethics Committee of Shanghai East Hospital. Patient data was collected anonymously, and informed consent was not required.

### 2.2 Inclusion and exclusion criteria

Eligibility criteria was delineated as follows: (1) Individuals of any age or gender. (2) Specific diagnostic parameters for pulmonary infections encompassed the identification of new or worsening focal or diffuse infiltrates on chest X-ray or computed tomography (CT), in conjunction with clinical symptoms such as fever, cough, increased sputum production, dyspnea, and hemoptysis. (3) Collection of a sufficient quantity of BALF sample (>20 ml) was mandatory.

Conversely, the exclusion criteria was as follows: (1) Sample-related issues: BALF samples failing to meet the NTS standards for quality control and testing procedures. (2) Patients with incomplete medical records.

### 2.3 Sample collection and laboratory testing

All patient underwent a bronchoscopy procedure. BALF selection was guided by the lesion sites identified on chest CT scans, followed by comprehensive testing of the BALF sample by CMTs, NTS, and other routine diagnostic modalities.

### 2.4 Establishment of the final clinical diagnosis

The final clinical diagnosis of each participant was used as the reference point for evaluating the sensitivity and specificity of the detection methods. The diagnostic results were determined at the time of patients discharge, following consultations among two respiratory physicians and a radiologist from the medical team. Patients were classified based on established clinical guidelines (Qu and Shi, 2018; Qu and Cao, 2016; Olson and Davis, 2020; Kalil et al., 2016), and the specific criteria for each condition are detailed below.

Community-Acquired Pneumonia (CAP) Diagnostic Criteria:

1. Community-acquired onset;
2. The clinical manifestations of pneumonia include the following: (1) Newly onset cough, sputum production, or exacerbation of pre-existing respiratory symptoms, with or without purulent sputum, pleuritic chest pain, dyspnea, or hemoptysis; (2) Fever; (3) Physical signs of consolidation or auscultatory findings of moist crackles; (4) Peripheral blood leukocyte count >10*10^9^/L or <4*10^9^/L, with or without neutrophilic left shift;
3. Chest imaging reveals the following: New onset of patchy infiltrates, consolidation, ground-glass opacities, or interstitial changes, with or without pleural effusion.

The clinical diagnosis of CAP is established if criteria 1, 3, and any one of the clinical manifestations in point 2 are met, after excluding alternative diagnoses such as pulmonary malignancy, non-infectious interstitial lung disease, pulmonary edema, atelectasis, pulmonary embolism, eosinophilic pneumonia, and vasculitis.

The diagnostic criteria for hospital-acquired pneumonia (HAP) are as follows:

HAP: Pneumonia developing after 48 hours of hospitalization in patients who have not received invasive mechanical ventilation.

Radiographic findings: Chest radiography or computed tomography (CT) showing new or progressive infiltrates, consolidation, or ground-glass opacities;

At least two of the following clinical signs: Fever (temperature >38°C; Purulent respiratory secretions; Peripheral blood leukocyte count >10*10^9^/L or <4*10^9^/L.

In cases where a definitive diagnosis could not be reached, the patient was categorized as “unknown.”

### 2.5 Conventional microbial testing

BALF samples were subjected to routine microbial tests, including bacterial, fungal, and mycobacterial cultures, acid-fast staining for mycobacteria, immunofluorescence staining for fungi, as well as assessments for *Aspergillus* galactomannan antigen and *Cryptococcus* antigen. Furthermore, T-SPOT or Xpert tests were selectively performed in patients suspected of having tuberculosis infections. Real-time PCR was utilized for detecting SARS-CoV-2, *Mycoplasma pneumoniae*, *Chlamydophila pneumoniae*, *cytomegalovirus* (CMV), adenovirus, *influenza A/B viruses*, *respiratory syncytial virus*, *human metapneumovirus*, and other pathogens. Serum samples were also screened for (1,3)-β-D-glucan, galactomannan, and *Cryptococcus* antigen.

### 2.6 Nanopore-targeted sequencing

#### 2.6.1 Nucleic acid extraction

Total nucleic acid extraction of samples and amplification methods were performed based on previously reported methods but with some modifications (Wang et al., 2020b; Neuenschwander et al., 2020). Briefly, all BALF samples were centrifuged at 20,000 × g for 10 min. The supernatant was removed and 200 μL retained for DNA/RNA extraction. The total nucleic acid was extracted using the Sansure SUPRall DNA Extraction Kit, (Changsha, China) following the manufacturer’ protocol. At the same time, 200 μL Tris-EDTA buffer was added in the batch as the negative control for nucleic acid extraction (extraction control, ETC).

#### 2.6.2 Library preparation and sequencing

All primers used in this study were adopted from a previous study (Hong et al., 2023). The library was constructed using a multiplex PCR capture library construction kit (DG021-1, Dgensee, WuHan, China) according to the manufacturer’s protocol. A total of 100 ng of DNA from each sample was added to the amplification system, and for samples with less than 100 ng of DNA, the entire available DNA was used. During this process, two rounds of PCR were performed to enrich the target pathogen sequences. The specific flow cell used was the R9.4.1 flow cell (FLO-MIN106, Oxford Nanopore Technologies) and the library was sequenced using Oxford Nanopore GridION X5.

#### 2.6.3 Bioinformatic analyses

Bioinformatics analysis and pathogen determination was performed as described (Hong et al., 2023; Wang et al., 2020a). Basecalling was performed using the Oxford Nanopore GridION X5 platform, employing Guppy in high accuracy mode (ont-guppy-for-gridion v.1.4.3-1 and v.3.0.3-1; high-accuracy basecalling mode). Sequencing reads with undesired length (<200 nt or >2000 nt) or that exhibited low quality (Q <7) were filtered out of the raw data. Adaptor trimming and barcode demultiplexing were conducted using Porechop (v.0.2.4). The reads were initially aligned against the 16S rDNA/ITS database (16S rDNA/ITS from ftp://ftp.ncbi.nlm.nih.gov/refseq/TargetedLoci), and the retained reads were subsequently mapped against the virus database (Virus from http://ftp.ncbi.nlm.nih.gov/refseq/release/viral/) using BLASTn (v.2.9.0+). For the reads preliminary assigned to the same species, a consensus sequence was generated using Medaka (v.0.10.1). Then, the consensus sequence was remapped to the 16S rDNA/ITS/virus reference database, and the taxon with the best match was adopted as the final detection result for reads from the same species, as determined in the preliminary taxonomy assignment (Specific software parameters can be found in the **Supplementary File 1**). The criteria used to select these pathogens was as follows: (1) Filtering out closely related microorganisms. To minimize cross-species misalignments among closely related microorganisms, we applied penalties to reduce the Relative Abundance Per Million (RPM) of microorganisms that share a genus or family designation. A penalty of 10% and 5% was used for genus-level matches and family-level matches, respectively. For example, if *Escherichia coli* had an RPM of 100 and *Shigella sonnei* (belonging to the same Enterobacteriaceae family) had an RPM of 5, the RPM of *S. sonnei* would be adjusted to zero (Gu et al., 2021). (2) Filtering out contaminants from negative controls. The negative controls were to designed to filter out bacteria and fungi contaminants from NTS laboratory sampling and from the human normal flora. Organism information was retained only if their abundance was higher in samples than in controls. Subsequently, a list of organisms, referred to as the PCR-organism list, was used to eliminate contaminants introduced by PCR (Jing et al., 2021). A reportable list of clinical pathogens was set up from published reports which applied NGS to identify the pathogens (kariusdx.com/karius‐test/pathogens) (Blauwkamp et al., 2019). The final report for each sample was discussed individually with a clinical microbiology specialist and clinicians responsible for each patient.

### 2.7 Statistical analysis

Sensitivity, specificity, positive predictive values and negative predictive value with 95% confidence intervals were reported as absolute values. Statistical significance was determined with a threshold of a P value <0.05. Comparative analysis was conducted by the Wilcoxon signed-rank test, Pearson χ2 test, Fisher exact test, or the McNemar test for discrete variables, as deemed appropriate. Bootstrapping was employed to analyze small sample sizes. Data was analyzed using SPSS 19.0 and GraphPad Prism 8 software. The concordance between NTS and CMTs was analyzed for all samples, and diagnostic performance was assessed using PID and NPID samples. Further comparative analysis was performed between CMTs and NTS on pathogen-positive detection rates in the context of antibiotic exposure in the PID group, as well as an exploration of the potential clinical benefits of NTS.

## 3 Results

### 3.1 Basic characteristics of enrolled patients

A total of 180 patients were enrolled in this study, including 122 (67.78%) males and 58 (32.22%) females with a mean age of 68.92 years. Half of the patients were smokers or had a history of smoking. Most patients (69.44%, 125/180) had previously been exposed to antibiotic treatment (received within 72 h) prior to sampling. Among the 180 patients, 137 (76.11%) patients were categorized as having a pulmonary infection, with identifiable pathogens being the cause of their pneumonia. Within this subgroup of 137 patients, 100 had community acquired pneumonia, 37 had hospital acquired pneumonia. A second subgroup of 32 patients were confirmed to have non-infectious diseases and included various conditions such as lung cancer (n=8), pulmonary nodules (n=2), bronchitis (n=6), bronchial asthma (n=4), bronchiectasis (n=5), chronic obstructive pulmonary disease (n=4), pulmonary emphysema (n=1) and connective tissue disease-associated interstitial lung disease (n= 2). For the remaining 11 cases the final diagnosis remained unclear because the aetiology of their pneumonia could not be determined. Additionally, PID showed higher inflammatory markers compared to NPID, including elevated white blood cell counts (9.68 vs 7.18 *10^9^/L), neutrophil percentages (76.50% vs 68.53%), CRP levels (51.79 vs 37.21 mg/L), and PCT levels (3.033 vs 0.67 ng/mL), as well as lower lymphocyte percentages (14.38% vs. 21.31%) (**Table 1**). These results indicate a stronger systemic inflammatory response in PID compared to NPID.

**Table 1:** Baseline characteristics of 180 patients.

Characteristics	Value
Age (year), (mean ± SD)	68.92 ± 12.64
Smoke history, n (%)	93 (51.67%)
Sex, n (%)	
Male	122 (67.78%)
Female	58 (32.22%)
Antibiotic exposure before NTS, n (%)	
Total (Y / N)	125 (69.44%) / 55 (30.56%)
PID group (Y / N)	106 (58.89%) / 31 (17.22%)
NPID group (Y / N)	15 (8.33%) / 17 (9.44%)
Unknown group (Y / N)	4 (2.22%) / 7 (3.89%)
137 PID patients, n (%)	
Community acquired pneumonia	100 (72.99%)
Hospital acquired pneumonia	37 (27.01%)
32 NPID patients, n (%)	
Lung cancer	8 (25.00%)
Pulmonary nodules	2 (6.25%)
Bronchitis	6 (18.75%)
Bronchial asthma	4 (12.50%)
Bronchiectasis	5 (15.63%)
Chronic obstructive pulmonary disease	4 (12.50%)
Pulmonary emphysema	1 (3.13%)
Connective tissue disease-associated interstitial lung disease	2 (6.25)
Inflammatory Parameters (PID vs NPID)	
WBC (×10^9^/L)	9.68 vs 7.18
NP (%)	76.50 vs 68.53
Lymphocyte (%)	14.38 vs 21.31
CRP (mg/L)	51.79 vs 37.21
PCT (ng/ml)	3.033 vs 0.67

### 3.2 Comparative analysis of diagnostic performance between NTS and CMTs

**Figure 2:**
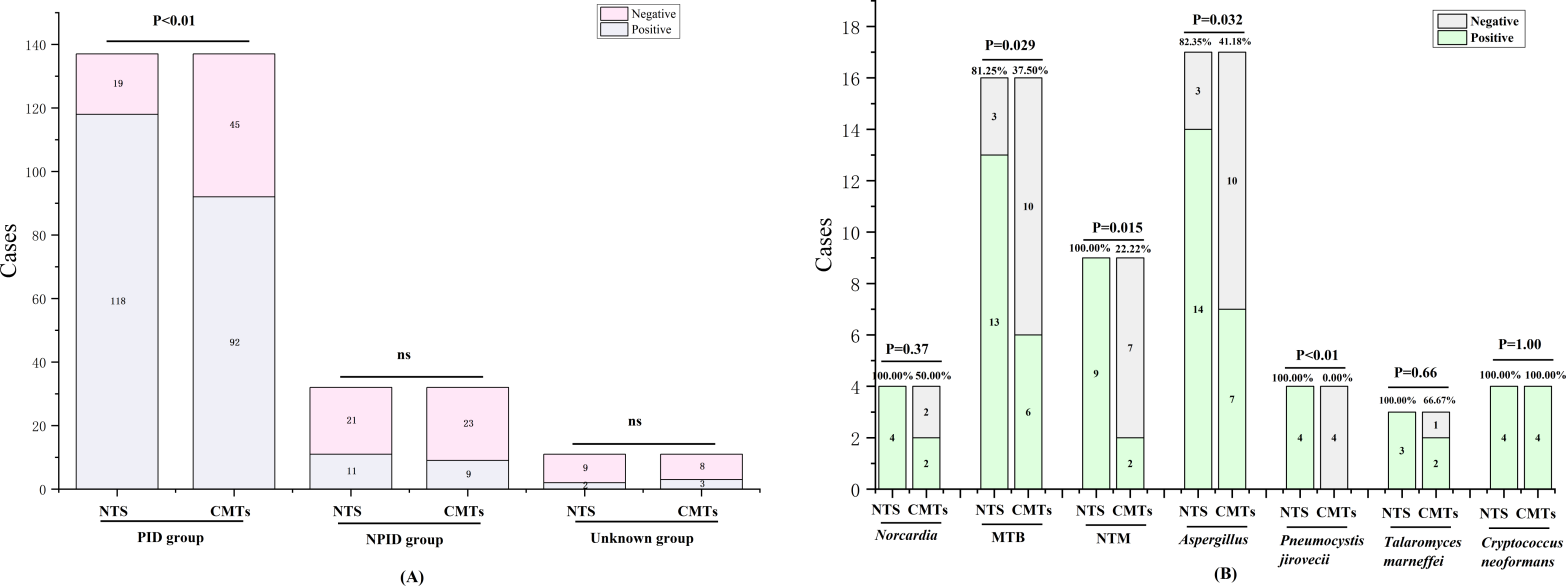
Positivity rate comparison analysis between NTS and CMTs. **(A)** Comparative analysis of positive rates between NTS and CMTs; **(B)** Comparison of diagnostic performance of NTS and CMTs for specific pathogens. ns, no significance.

In this study, the positive rates of NTS for PID, NPID, and unknown groups were 86.13% (118/137), 34.38% (11/32), and 18.18% (2/11); respectively, whereas the corresponding positive rates for CMTs were 67.15% (92/137), 28.13% (9/32), and 27.27% (3/11). Statistical analysis revealed a significant difference between the positive detection rates of NTS and CMTs in the PID group (P < 0.01), while no statistical difference was found between the NPID and unknown aetiology group (**Figure 2A**). Furthermore, we compared and analyzed the results of NTS and CMTs for detecting specific pathogenic microorganisms that pose diagnostic challenges, including *Mycobacterium tuberculosis* (MTB), non-tuberculous *Mycobacterium* (NTM), *Nocardia*, *Aspergillus*, *Talaromyces marneffei*, *Pneumocystis jirovecii*, and *Cryptococcus neoformans*. Among these, NTS showed significantly higher positive detection rates than CMTs for MTB (81.25% vs 37.50%, P=0.029), NTM (100.00% vs 22.22%, P=0.015), *Aspergillus* (82.35% vs 41.18%, P=0.032), and *P. jirovecii* (100.00% vs 0.00%, P<0.01) (**Figure 2B**). NTS detected two additional cases of *Nocardia* and one additional case of *T. marneffei* compared to CMTs, although these differences were not statistically significant. Furthermore, both NTS and CMTs identified four cases of *C. neoformans* (**Figure 2B**). Additionally, we compared the diagnostic efficiencies of NTS and CMTs for differentiating between PID and NPID in 169 patients. The sensitivity and specificity of diagnosing PID were 86.13% vs 67.15% (NTS VS CMTs, P < 0.01) and 65.63% vs 71.88% (NTS VS CMTs, P = 0.59), respectively; and the positive predictive values (PPVs) and negative predictive values (NPVs) were 91.47% vs 91.09% (NTS VS CMTs, P = 0.90) and 52.50% vs 33.82% (NTS VS CMTs, P = 0.056), respectively (**Table 2**).

**Table 2:** Diagnostic performance of NTS and CMTs in suspected pulmonary infectious patients.

Diagnostic testing	Results	PID	NPID	Sensitivity % (95% CI)	Specificity % (95% CI)	PPVs % (95% CI)	NPVs % (95% CI)
NTS	+	118	11	86.13 (79.27-91.02)	65.63 (48.23-79.67)	91.47 (85.23-95.31)	52.50 (37.50-67.07)
	–	19	21				
CMTs	+	92	9	67.15 (58.90-74.47)	71.88 (54.46-84.60)	91.09 (83.74-95.43)	33.82 (23.68-45.69)
	–	45	23				
P value				<0.01	0.59	0.90	0.056

### 3.3 Diagnostic concordance between NTS and CMTs

**Figure 3:**
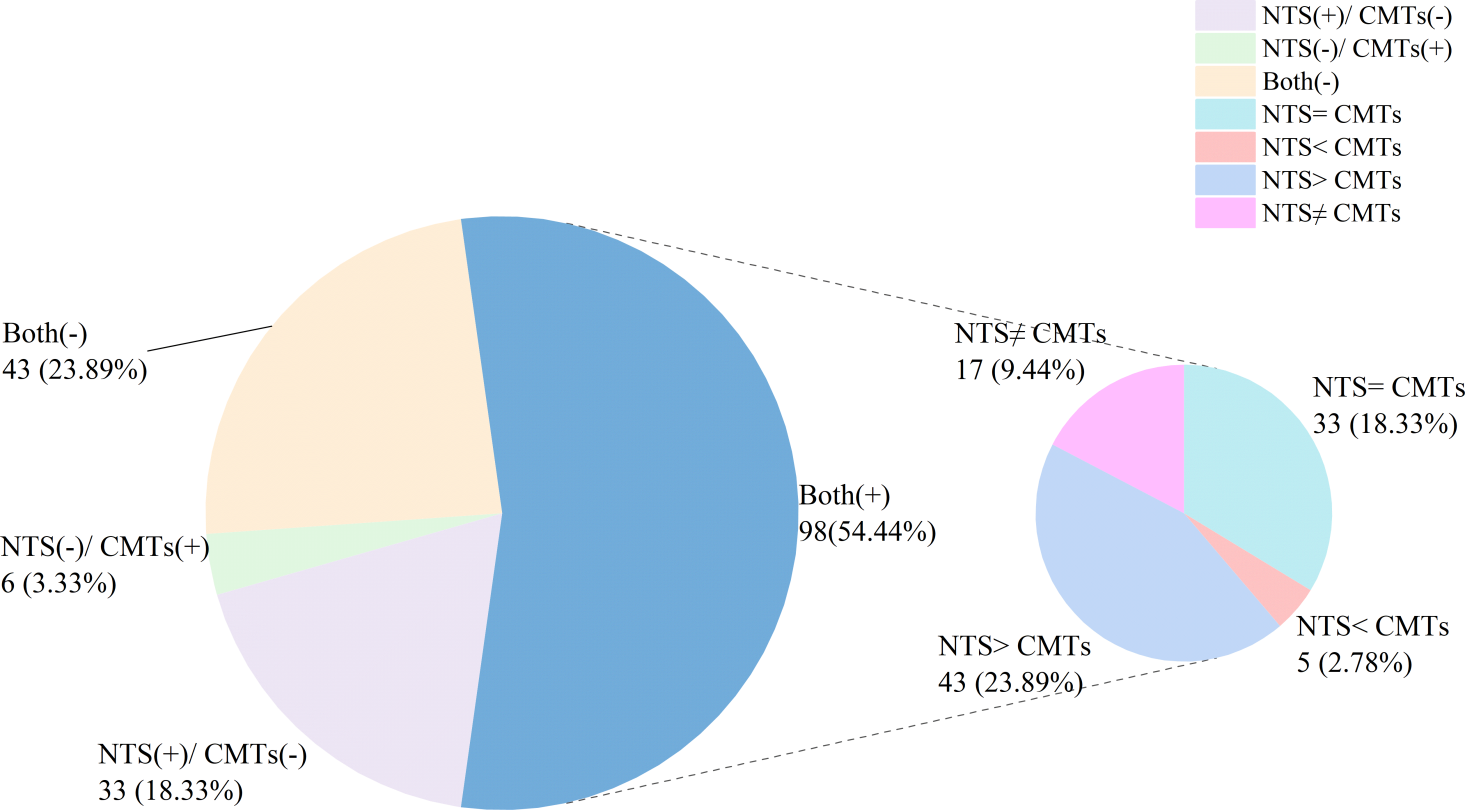
Concordance analysis between NTS and CMTs.

In this study, 43 (23.89%) cases had negative results for both NTS and CMTs, while 98 (54.44%) cases showed positive results for both (**Figure 3**). Among the remaining cases, 33 (18.33%) were positive for NTS but negative for CMTs whereas only 6 (3.33%) were negative for NTS but positive for CMTs (**Figure 2**). Of the 98 (54.44%) cases with positive results for both NST and CMTs, there were complete concordance (NTS = CMTs) in 33 (18.33%) cases, while 43 (23.89%) cases showed NTS results fully covering the culture results (NTS > CMTs), 5 (2.78%) cases showed culture results fully covering NTS results (NTS < CMTs), and 17 (9.44%) cases showed complete non concordance between the two testing methods (NTS ≠ CMTs) (**Figure 3**).

### 3.4 Analysis of inconsistent results between NTS and CMTs

Comparing to CMTs results, NTS identified additional 138 pathogens. These pathogens identified by NTS included *Actinobacillus pleuropneumoniae* (n = 1), *Clostridium perfringens* (n = 1), *Corynebacterium striatum* (n = 1), *Morganella morganii* (n = 1), *Norcardia* (n = 2), *Staphylococcus* (n = 3), *Achromobacter xylosoxidans* (n = 3), *Enterobacter cloacae* (n = 3), *Escherichia coli* (n = 3), *Enterococcus* (n = 4), *Elizabethkingia anopheles* (n = 4), *Streptococcus* (n = 5), *Stenotrophomonas maltophilia* (n = 5), *Haemophilus* (n = 5), *Serratia marcescens* (n = 5), *Klebsiella* (n = 6), MTB (n = 7), NTM (n = 7), *Pseudomonas* (n = 8), *Acinetobacter* (n = 11), *Cladosporium cladosporium* (n = 1), *Aureobacidium pullulans* (n = 1), *T. marneffei* (n = 1), *P. jirovecii* (n = 5), *Aspergillus* (n = 8), *Candida* (n = 10), *Human herpesvirus* 7 (HHV-7) (n = 1), *Torque teno virus* (TTV) (n = 1), *Cytomegalovirus* (CMV) (n = 6), and *Herpes simplex virus* 1 (HSV-1) (n = 19) (**Table 3**). Through comprehensive analysis by two senior medical experts, the 56 of the 138 pathogens were considered consistent with the clinical presentation, including *Norcardia* (n = 2), *Staphylococcus* (n = 3), *E. cloacae* (n = 1), *Enterococcus* (n = 1), *E. anopheles* (n = 1), *Streptococcus* (n = 1), *S. maltophilia* (n = 1), *Haemophilus* (n = 2), *S. marcescens* (n = 1), *Klebsiella* (n = 4), MTB (n = 7), NTM (n = 7), *Pseudomonas* (n = 5), *Acinetobacter* (n = 5), *P. jirovecii* (n = 4), *Aspergillus* (n = 8), CMV (n = 1), and HSV-1 (n = 2) (**Table 3**). Among the 35 negative NTS results but with positive CMTs results, most of the pathogens were detected by NTS but did not meet the criteria for positive reporting. There were only 9 cases where there were no pathogens detected, and 1 of these cases was suspected to have sample contamination during culture (**Table 3**).

**Table 3:** Inconsistent results between CMTs and NTS.

Additional pathogens detected by NTS	The number of cases	Consistent with the clinical presentation	Pathogens not reported by NTS	The number of cases	Possible explanation		
					Not detected	Detected but not meeting positive criteria	Contamination
*Actinobacillus pleuropneumoniae*	1	0	*Citrobacter freundii*	1	1		
*Clostridium perfringens*	1	0	*Enterobacter cloacae*	1		1	
*Corynebacterium striatum*	1	0	*Escherichia coli*	1		1	
*Morganella morganii*	1	0	*Stenotrophomonas maltophilia*	1		1	
*Norcardia*	2	2	*Acinetobacter baumannii*	1		1	
*Staphylococcus*	3	3	*Talaromyces aculeatus*	1			1
*Achromobacter xylosoxidans*	3	0	*Aspergillus*	1	1		
*Enterobacter cloacae*	3	1	RSV^7^	1	1		
*Escherichia coli*	3	0	*Staphylococcus aureus*	2	2		
*Enterococcus*	4	1	*Penicillium oxalicum*	2		2	
*Elizabethkingia anopheles*	4	1	*Klebsiella*	3	3		
*Streptococcus*	5	1	*Pseudomonas aeruginosa*	3	1	2	
*Stenotrophomonas maltophilia*	5	1	*Corynebacterium striatum*	4		4	
*Haemophilus*	5	2	*Candida*	13		13	
*Serratia marcescens*	5	1	Total	35	9	25	1
*Klebsiella*	6	4					
MTB^1^	7	7					
NTM^2^	7	7					
*Pseudomonas*	8	5					
*Acinetobacter*	11	5					
*Cladosporium cladosporium*	1	0					
*Aureobacidium pullulans*	1	0					
*Talaromyces marneffei*	1	0					
*Pneumocystis jirovecii*	5	4					
*Aspergillus*	8	8					
*Candida*	10	0					
HHV-7^3^	1	0					
TTV^4^	1	0					
CMV^5^	6	1					
HSV-1^6^	19	2					
Total	138	56					

MTB^1^, Mycobacterium tuberculosis; NTM^2^, Non-tuberculosis mycobacteria; HHV-7^3^, Human herpesvirus 7; TTV^4^, Torque teno virus; CMV^5^, Cytomegalovirus; HSV-1^6^, Herpes simplex virus 1; RSV^7^, Respiratory Syncytial Virus.

### 3.5 Antibiotic exposure and pathogen detection

**Figure 4:**
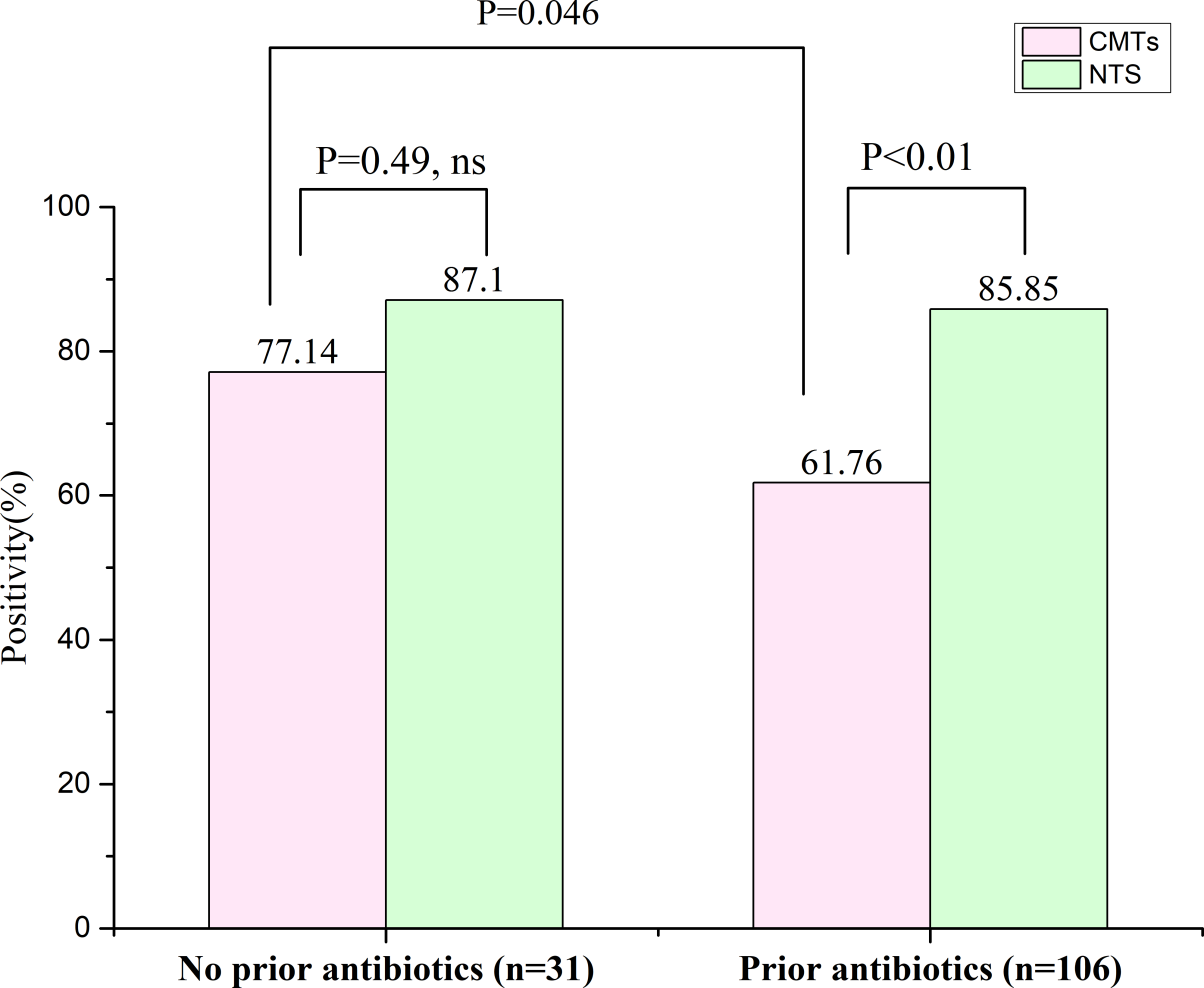
Effect of previous antibiotic exposure on the positivity rate of NTS in the PID cases. ns, no significance.

A total of 137 confirmed PID patients were included in this study, of whom 106 had a history of antibiotic exposure and 31 no exposure. Among those with antibiotic exposure, the positive detection rates for NTS and CMTs were 85.85% (91/106) and 61.76% (65/106), respectively; and the difference was significant (P < 0.01) (**Figure 4**). In contrast, among those without antibiotic exposure, the positive detection rates for NTS and CMTs were 87.10% (27/31) and 77.14% (25/31), respectively, with no significant differences observed (**Figure 4**). Furthermore, the positive rate of CMTs before antibiotic exposure was 77.14%, whereas after exposure, it decreased to 61.76%, a significant difference (P = 0.046) (**Figure 4**). It is worth noting that the positive detection rate of NTS was significantly less affected by previous antibiotic exposure compared to CMTs.

### 3.6 Adjustment and effectiveness of antibiotic treatment after NTS

**Figure 5:**
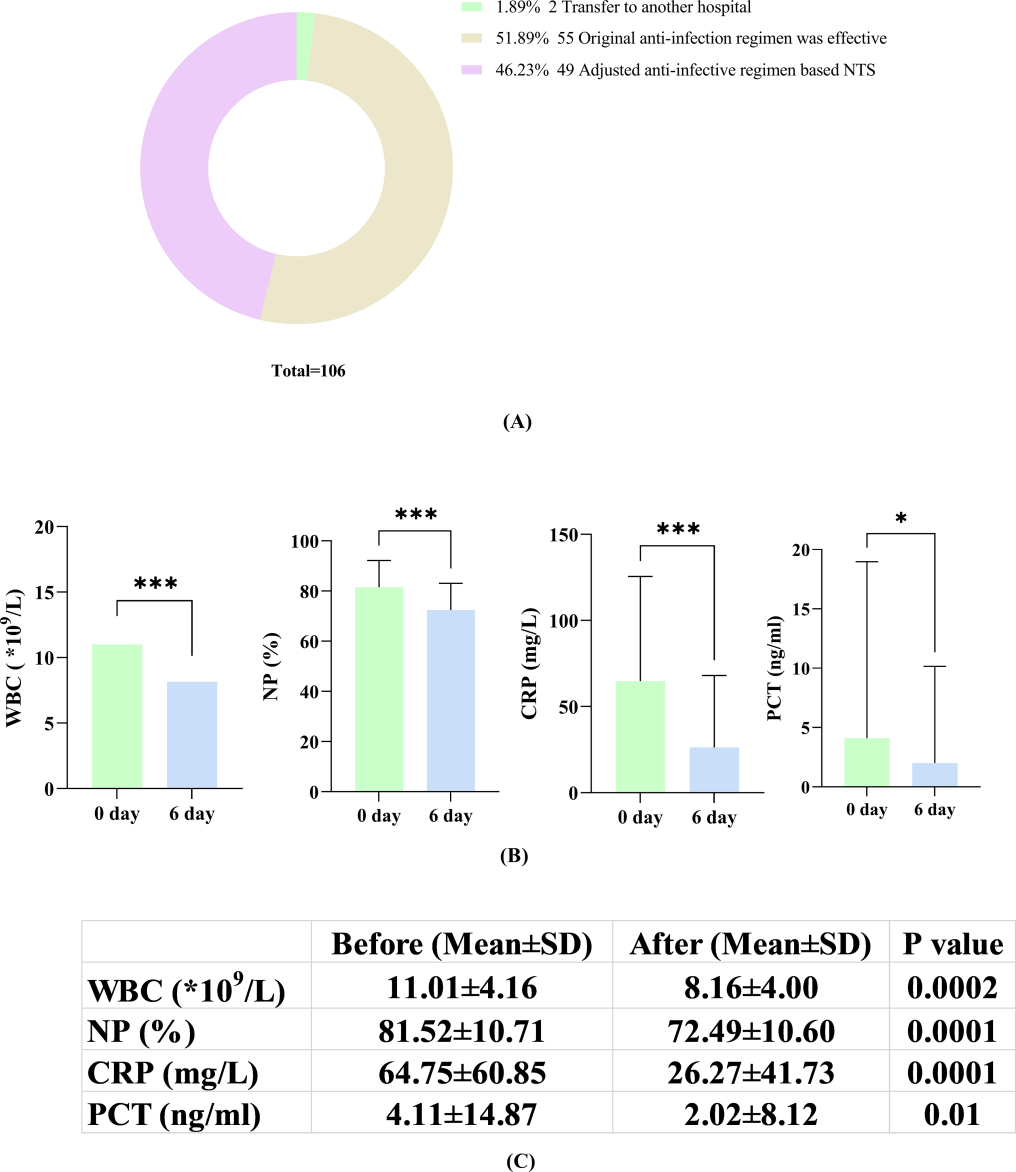
**(A)** Adjustment of antibiotics according to the NTS results; **(B)** Changes in inflammatory markers (WBC, NP, CRP and PCT) on the day of NTS versus 6 days after adjustment based on NTS; **(C)** Comparison of patients on the day of NTS and on the 6th day after the adjustment of antibiotics according to NTS results. *, P<0.05; ***, P<0.001.

As shown in **Figure 4**, among 106 individuals in the PID group with prior antibiotic use, 55 retained their prior regimen due to its effectiveness. Two patients were transferred to other facilities, making infection control efficacy unclear. Furthermore, 49 patients underwent adjustments to their antibiotic prescriptions after receiving NTS test reports in the clinic (**Figure 5A**). Within this subset of 49 patients, 43 (43/49, 87.76%) exhibited improvement in their condition with the change in antibiotic regimen, while only 6 (6/49, 12.24%) did not show significant alleviation of symptoms. The average time for symptom relief in these patients was 6 days (± 2.3 days). Comparison of blood inflammation indicators between the day of the NTS test and 6 days after adjusting antibiotics revealed notable decreases in white blood cell (WBC), neutrophil percentage (NP), C-reactive protein (CRP), and procalcitonin (PCT) levels. Specifically, prior to medication adjustment, the values were 11.01 ± 4.16 (*10^9^/L), 81.52 ± 10.71 (%), 64.75 ± 60.85 (mg/L), and 4.11 ± 14.87 (ng/ml), respectively. After antibiotic adjustment, the values were 8.16 ± 4.00 (*10^9^/L), 72.49 ± 10.60 (%), 26.27 ± 41.73 (mg/L), and 2.02 ± 8.12 (ng/ml), respectively, demonstrating a clear downward trend in all parameters (**Figures 5B, C**). Detailed data for these indicators are provided in the **Supplementary file 2**.

### 3.7 Turnaround time of nanopore sequencing and analysis in clinical practice

**Figure 6:**
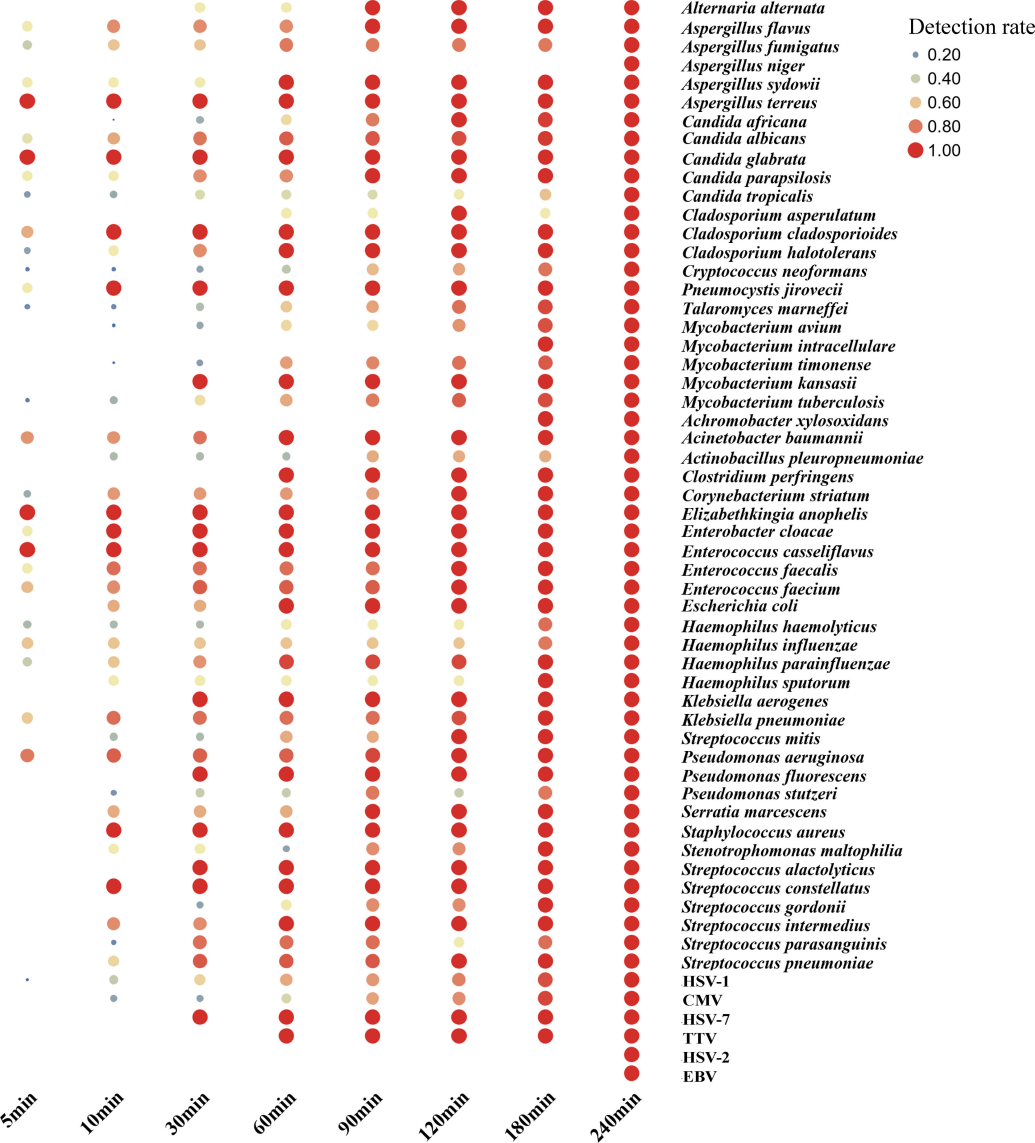
Nanopore sequencing real-time analysis for the detection rate of gram-positive/negative bacteria, virus and other critical pathogen in this study. In this cohort, from the initiation of sequencing, it is observed that 47%, 75%, 87% and 95% of the pathogens were detected within 5 min, 10 min, 30 min and 180 min, respectively.

Nanopore sequencing enables real-time analysis, and the study involved calculating the time required for analyzing all samples from sequencing initiation to delivery of an interpretable result. Our findings indicated that 47% of pathogens could be detected within 5 minutes, with detection rates increasing to 75%, 87%, and 95% within 10, 30, and 180 minutes, respectively. Notably, key pathogens like *Aspergillus terreus*, *Acinetobacter baumannii*, and *Pseudomonas aeruginosa* exhibited over 70% positive detection within 5 minutes. In the case of MTB detection, a 180-minute timeframe was necessary to detect 91% of positive cases. Regarding virus detection, a minimum of 10 minutes was required to detect high-abundance HSV-1 and CMV. However, the positive detection rate for HSV-2 and *Epstein-Barr virus* (EBV) was only 20-40%, necessitating over 240 minutes for detection (**Figure 6**).

## 4 Discussion

PID is a common respiratory disease that poses a major threat to human health worldwide. The timely and effective identification of infectious pathogens is critical for reducing morbidity and mortality. However, traditional methods are often inadequate for comprehensively and effectively identifying pathogens within a short time frame.

NTS was developed by amplifying the 16S rRNA gene (for bacteria), ITS1/2 gene (for fungi), or specific viral genes (for viruses) and using long read sequencing of the amplified marker genes. Several studies have described the performance of NTS in the diagnosis of infectious diseases (Zhang et al., 2023; Hong et al., 2023; Fu et al., 2022; Huang et al., 2021). In the study that analyzed different specimen types such as pleural and ascites fluid, bronchoalveolar lavage fluid, cerebrospinal fluid, urine, blood and wound secretions, it found that the overall detection sensitivity of NTS (94.5%) was greatly increased by 56.7% compared with culture (37.8%) (Hong et al., 2023). In the other studies, NTS had a significantly higher positive detection rate of bloodstream infections in neutropenic patients than in blood cultures (63.36% vs 14.85%) (Hong et al., 2023)and NTS was significantly more sensitive than bacterial culture in the diagnosis of infectious endophthalmitis (94.44% vs 44.44%) (Huang et al., 2021). In our retrospective study, we conducted a systematic comparison and analysis of the diagnostic capabilities of CMTs and NTS for detecting PID. Our findings demonstrated that NTS exhibited a significantly higher etction sensitivity (86.13%) than CMTs (67.15%) for PID, particularly in the case of specific pathogens, such as MTB, NTM, *Aspergillus*, and *P. jirovecii* (**Figure 2**). Additionally, NTS was able to detect two additional cases of *Nocardia*, although the difference was not significant due to the limited sample size. The detection of the listed bacterial pathogens, such as *P. aeruginosa*, *A. baumannii*, *C. cladosporioides*, *Enterococcus faecium*, *Haemophilus influenzae*, and *Klebsiella pneumoniae*, as well as MTB, was feasible within 5 minutes using the NTS method. However, virus detection typically require more time compared to bacteria and fungi detection. One reason for this disparity is the complexity of viral genomes, which can vary significantly in size and structure, making their identification and analysis more intricate and time-consuming. Additionally, viruses may exist in lower concentrations within samples, necessitating more sensitive detection methods and potentially longer sequencing times for accurate identification. Nevertheless, this finding highlights the potential of NTS in detecting a wider range of microorganisms than CMTs in a faster timeframe, and is consistent with previous studies (Fu et al., 2022; Zhang et al., 2023).

We analyzed discrepancies in the results obtained from the two methods using CMTs, which found that NTS results additionally reported 138 positive pathogens and 35 pathogens were only detected by CMTs (**Table 3**). Based on a comprehensive analysis by two senior medical experts, it was found that most of the additional reported pathogens were likely identified as environmental background microorganisms, but 56 pathogens of them were considered clinically relevant (**Table 3**). As for the pathogens only detected by CMTs, 1 case was due to contamination during the cultivation process, 25 cases were detected by NTS but did not meet the reported criteria, and only 9 cases were actually not detected by NTS (**Table 3**). Thus, in terms of microbial detection, the results of NTS can essentially replicate those of CMTs, but also identify a greater number of microbes. How do we rationalize these differences? Firstly, some bacteria, such as *Streptococcus pneumoniae* and *H. influenzae* are easily mistaken for other colonizing bacteria during culture identification, leading to missed detection. Additionally, during cultivation, the growth of certain non-dominant pathogens can be suppressed by nutritional competition, resulting in false negatives. Moreover, some slow-growing or even non-cultivable pathogens, such as MTB, NTM, and *P. jirovecii* are challenging to detect using traditional methods, whereas NTS exhibits superior detection capabilities. Lastly, NTS detects pathogenic nucleic acids, while CMTs primarily focuses on detecting live pathogens. Therefore, any factors affecting pathogen activity may interfere with the efficiency of CMTs detection. For instance, in this study, the positive rate of CMTs before antibiotic exposure was 77.14%, but in patients exposed to antibiotics, the positivity rate dropped to 61.76%. In contrast, the corresponding positive detection rates for NTS were 87.10% and 85.85%, respectively (**Figure 4**). Despite the limited sample size, these findings suggest that NTS may be less affected by antibiotic exposure than CMTs when detecting pathogens. Additionally, 49 patients from 106 PID cases who received standard antibiotic treatments treatment had their prescriptions adjusted based on NTS results (**Figure 5A**). Among these, 43 (87.76%) patients showed improvement, 6 (12.24%) did not experience significant symptom alleviation. The average time for symptom relief was around 6 days (± 2.3 days). Analysis of blood inflammation indicators before and after antibiotic adjustment revealed a notable overall decrease in WBC, NP, CRP, and PCT levels (**Figures 5B, C**). These findings suggest that NTS can play a positive role in guiding clinical practice and holds significant reference value for optimizing the treatment of pulmonary infections.

The NTS testing protocol, which involves approximately 0.5 hours for sample processing and nucleic acid extraction, as well as 3 hours for library preparation, enables 95% of samples to be analyzed and results reported within 6 hours. Furthermore, in urgent scenarios, a single clinical sample can be expedited for testing alongside quality control samples. For samples with a higher microbial load, NTS can promptly identify and report pathogens within the initial 5 minutes of sequencing, thus delivering vital microbiological evidence essential for the prompt and precise treatment of critically ill patients. The whole turnaround time of NTS is much shorter than that of traditional culture methods, which range from 2–3 d for bacteria, 2–7 d for fungi, and 45–60 d for mycobacteria. Thus, NTS is a fast and cost-effective sequencing technology that can add clinical value to traditional methods of infectious disease testing.

Nevertheless, it is important to note that the current NTS method used in this research has some limitations. For example, the test is based on pathogen detection only and does not include parasite targets. This decision was made because pulmonary parasitic infections are rare in modern China, and the current panel is tailored to the local epidemiological context. However, with the targeting design format, it will be simple to include antibiotic resistance detection which will provide more information on the nature of bacterial infections and allow more personalized antibiotic administration to effectively treat the infection. In addition, NTS, like other sequencing technologies, is susceptible to the effects of background host nucleic acids and amplification inefficiency, and the abundance of amplified reads may not truly directly reflect the composition of pathogenic organisms in the original sample. Another limitation is that this study was conducted at a single center, which may restrict the generalizability of the findings to other clinical settings. While this approach is useful in the context of the local hospital, broader multicenter studies will be necessary to confirm its applicability across different populations and regions.

Our study primarily focuses on nanopore sequencing and conventional methods. However, we plan to incorporate mNGS results in future research to expand the diagnostic applications and improve microbial detection accuracy.

## 5 Conclusions

In conclusion, our research highlights current clinical utility of the recently introduced NTS technique, enabling a rapid and precise diagnosis of pulmonary infections from bronchoalveolar lavage fluid samples. Although NTS cannot completely replace traditional testing methods, it can serve as a valuable and reliable tool to assist in the clinical diagnosis of challenging cases.

## Data Availability

The raw sequence data reported in this paper have been deposited in the Genome Sequence Archive (Chen et al., 2021) in National Genomics Data Center (Members and Partners, 2023), China National Center for Bioinformation / Beijing Institute of Genomics, Chinese Academy of Sciences (GSA: CRA010916) that are publicly accessible at https://ngdc.cncb.ac.cn/gsa/browse/CRA010916.
