# Supplementary material for: Clinical utility of nanopore-targeted sequencing for diagnosing and treating pulmonary infectious diseases from bronchoalveolar lavage fluid
Source: Front Cell Infect Microbiol. 2025 Feb 19;15:1469440. doi: 10.3389/fcimb.2025.1469440 (PMC11879936; doi:10.3389/fcimb.2025.1469440)
Supplement: Supplementary file 1 [file DataSheet1.docx]

Bioinformatics pipeline details:

Step1：Sequencing reads with undesired length (<200 nt or >2000 nt) or that exhibited low quality (Q <7) were filtered out of the raw data by an in-house python scripts.

Step2: Adaptor trimming and barcode demultiplexing were conducted using Porechop (v.0.2.4) with the parameters (require_two_barcodes:True, barcode_threshold:85).

Step3: The reads were aligned by BLASTn (v.2.9.0+). Reads mapped against 16S rDNA/ITS reference database of each sample were filtered by coverage >90% and E value =1e-5. Reads mapped against virus reference database of each sample were filtered by identity ≥90% and E value =1e-5. Then, the taxonomy of each read was assigned according to the taxonomic information of the mapped subject sequence.

Step4: For the reads preliminary assigned to the same species, a consensus sequence was generated using Medaka (v.0.10.1) with the default parameters.

Step5: The consensus sequence mapped against 16S rDNA/ITS reference database of each sample were filtered by coverage >90% and E value =1e-5. The consensus sequence mapped against virus reference database of each sample were filtered by identity ≥90% and E value =1e-5.
